# “With the Pandemic Still Raging, I am Blessed to Do My Part to Defeat it”: Exploring COVID-19 Jewish Liturgy and Prayers in Israel and the United States

**DOI:** 10.1007/s10943-024-02190-6

**Published:** 2024-12-04

**Authors:** Elazar Ben-Lulu

**Affiliations:** Ariel University, Ariel, Israel

**Keywords:** Body, Jewish liturgy, Prayer, COVID-19, Jewish communities

## Abstract

During the COVID-19 pandemic, synagogues faced closure, and many non-Orthodox communities transitioned their prayer services to online platforms. This presented a significant challenge for community leaders and rabbis who were faced with a profound community crisis. An innovative response emerged including new prayers and the adaptation of existing ones to better address the pandemic’s unique realities, integrating aspects of health, divinity, community, and the environment. This study engages in a textual analysis of these prayers, exploring how these mirror cultural and social attitudes toward the body and embodiment. While the body was seen during the COVID-19 period as problematic, an object of contagion and spreader of disease (e.g., by not keeping proper distance or masking), in these particular texts it is no longer slandered, but revealed as an obedient and disciplined agent. The prayers seek to overcome the disruption in the individual’s relationship with their body and with other bodies. The prayer authors propose to the worshipper, while also conceptually changing traditional ideas and practices, to view the body as an object that must be cleaned, vaccinated, purified, and allowed to continue its function. The concern for both the well-being of the living body and the dignity of the deceased extends to care for society and humanity as a whole. Therefore, this liturgy can be seen as a pragmatic means to promote a “theology of humanistic responsibility.”

## Introduction

Throughout Jewish history, prayer has held a central position in shaping Jewish identity, fostering community and nurturing faith. In addition to serving as a direct conduit for strengthening one’s relationship with the Creator, prayer also serves as a means of connection to self and community. Engaging in prayers, supplications, and encounters with sacred texts has been observed to have a therapeutic impact, contributing to healing and promoting mental well-being (Sered 1996, Guzmen-Carmeli & Rubin, 2014).

The act of prayer brings structure and significance to time and space in which it takes place (Giordan & Woodhead, 2017; Wittberg, 2013). As a result, Jews have developed various prayers and blessings at different times and various locations, enabling them to shape and validate their narrative, ethos, and faith. Through this understanding, Jews grasp that prayer in Judaism is both a ritualistic and textual domain imbued with power dynamics that evolve and adapt, reflecting and simultaneously establishing social order. In the well-known piyyut (liturgical poem) “Unetanneh Tokef,” recited in many communities during Rosh Hashanah and Yom Kippur, it is stated: *“And repentance, prayer, and charity can mitigate/annul the severity of the decree.”* Does prayer possess the power to mitigate the decree, to transform the dystopian reality into a utopian reality and to restore order to the world? Perhaps the elusive answer lies in simply engaging in prayer itself.

This discussion explores how various prayers created during the COVID-19 pandemic sought to provide a religious and spiritual response to the prevailing circumstances: the new restrictions and behavioral norms and the unprecedented situations that impacted customs and religious practices. Specifically, this paper focuses on the body and embodiment expressions, which played a central role in the emergence and spread of the virus, infecting many individuals. It aims to explore the body’s positioning within prayer, the embodied attitudes toward it, the demands and appeals made to the body, and to gain insights into our cultural and social perceptions regarding our own bodies.

Epidemics, plagues, and infectious diseases have significantly influenced Jewish history. The Talmudic Sages viewed infectious diseases as integral to God’s created world. Throughout the premodern and early modern eras, from plagues to diseases like smallpox and cholera, these periods prompted exploration of the Jewish community’s relationship with wider society. For instance, during the Black Death in 1348, Jews suffered both from the disease itself and from anti-Semitic violence sparked by blame (Brown, 2023b).

Textual analysis of several COVID-19 prayers shows how these particular texts shed light on the varied cultural responses and provides perceptions of the body during times of crisis and also within the context of normalcy. Thus, the paper exposes the way in which the attitude toward the body is revealed and reflects the attitude toward society itself. The body is the site through which society reflects and constructs its values, preserves, or changes them in the anomalous reality of an epidemic.

As the following descriptions illustrate, this analysis demonstrates how COVID-19 prayers manifest as a cultural textual pattern that reshapes social relations between healthy and sick bodies, as well as between healthy and deceased bodies. In these particular texts, which sometimes arise or is created through the de-conceptualization of traditional practices, the body undergoes a transformation from a maligned entity to a compliant and disciplined agent. The prayers aim to rectify the disturbance in the individual’s connection with their body and with others, promoting a sense of supervision and propriety. Through prayer, the body is seen as needing cleansing, vaccination, and purification to maintain its function. This concern for bodily health also extends to overseeing individuals’ behaviors or medical decisions, such as whether to receive vaccinations, reflecting a broader concern for the well-being of society and humanity.

In addition, it also highlights human responsibility that transcends borders of nationality or religion and prompts reexamination of the individual’s relationship with faith and God. While recent research has scrutinized the balancing of individual rights with governmental policies during epidemics, such as the tension between public health protection and privacy rights (Cohen-Almagor & Haber, 2023) or the setting of new educational programs (Kaiser et al., 2021), this study highlights how the religious sphere, particularly prayer, can foster self-health habits and encourage both personal and collective responsibility.

### Creative Ritualism: Religious Responses to the Pandemic

Throughout Jewish history, new prayers have been created with relative frequency, while old prayers have been rejuvenated, re-interpreted and imbued with new meanings to reflect the transformations and experiences of individuals within the community. Event-specific prayers as well as spontaneous prayers, which emerge as responses to circumstances of specific time and place, have been created and long coexisted with regular and obligatory prayers. Although, from a halakhic perspective, these prayers do not possess a fixed and codified status, they express the immediate needs, joys, pains, and anxieties of the Jewish people in direct and unfiltered ways (Marx, 2006). Additionally, they may also reflect the theological and moral perspectives of their creators, their attitudes toward various physical practices, cultivation of consciousness regarding prohibition, obedience, and also the examination of relationships between the Jewish person and life, and between the Jewish person and death.

Prayers composed during specific events or time periods provide insights into the Jewish experience, which may reflect a blending of local customs and prayer traditions. However, prayer is not solely a communal experience that takes place within a group; it is also an intimate moment where individuals connect with God and even themselves. Prayer allows worshippers to express gratitude and make requests, serving as a necessary mediator between the mundane and the divine.

In contrast to Schweid (2022), who defined prayer as a moment in time where the usual rules and norms do not apply to worshipers, I demonstrate how prayer serves as a mechanism for ensuring behavioral conformity concerning the worshiper’s bodily actions during the pandemic. Prayer is thus deployed as a means of monitoring and controlling behavior to mitigate the spread of the virus.

Through prayer, the individual seeks to make sense of their reality, sometimes stemming from a place of acceptance and introspection and at other times from a plea or even defiance. Prayer is not contingent on religious observance in an institutional sense or adherence to dogmatic beliefs. Rather, it is shaped by the sincerity and integrity of one’s heart (Sagi 2006).

Rabbi Abraham Joshua Heschel (1954) emphasized that prayer serves as the primary means to express gratitude to God, stating: “The focus of prayer is not prayer itself; the focus of prayer is God. One cannot pray without having faith in their own ability to turn to an eternal, infinite, and compassionate God” (P.44). Relatedly, Derrida (2013) argued that prayer should be seen as an act rather than a statement of truth or falsehood. In prayer, there exists a willingness to relinquish the expectation that the request within it will be answered or even heard; this reduces the anthropomorphism of the Creator. This study focuses on how during a pandemic, characterized by fear and uncertainty, COVID-19 prayers may manifest an epistemological tension between uncertainty and skepticism, as well as the decision to take personal and social responsibility (such as choosing to get vaccinated).

Similar to other non-Jewish religious group responses to the pandemic (Addo, 2021; Adichie, 2021; Budaev, 2021; Corpuz & Sarmiento, 2021; Davies, 2020; McCarthy, 2021), research on how the Jewish community has navigated COVID-19 and how it has influenced community structure, legal adaptations, and religious concepts is still ongoing. Some researchers view this period as an opportunity to understand how Judaism addresses crises and provides guidance across the spectrum, from secular to ultra-Orthodox individuals.

According to Langer (2021, p. 33), who examines diverse liturgical responses of Jewish communities to COVID-19, the possibilities for public prayer during the pandemic have been shaped by a community’s location on the spectrum of halakhic (Jewish Law) understanding, especially regarding the requirements for a minyan (prayer quorum) and the permissibility of using technology on Sabbaths and holy days. In other words, the place of Halakha and faith is still preserved as a central part of the adapted liturgical writing.

For instance, Epstein (2022), in her reflection on “Tefilat Le’et Hakorona” (“A Prayer in a Time of Corona”), shows how the author places faith as a central element in the foundation of the prayer, as the crying out for God’s salvation. However, this paper expands the conclusion about spirituality and may provide fertile ground for contemporary intersections and conjunctions between religiosity and medicine and body and society.

However, while previous articles have explored topics such as Jewish ethics (Rashi, 2020, 2021, 2022), the impact of virtual prayer practices (Kühle and Larsen 2021, Parish, 2020, Ben-Lulu 2021), mental health in conservative/traditional Jewish communities (Pirutinsky et al., 2020), attitudes toward vaccination and the healthcare system (Carmody et al., 2021), the relationship between Israeli ultra-Orthodox Jews and their rabbis (Cohen et al., 2021) and their processes of COVID-19 health decision-making (Taragin-Zeller et al., 2020), and the stigmatization of Jewish communities for non-compliance with guidelines (Gilman, 2021), this study will focus on the textual dimension.

Specifically, it will examine the role of prayers, their message, and how they express cultural and social perceptions of the body. In addition, it demonstrates the power of liturgical creativity and its adaptation to the reality of the pandemic; a process that allows for a relevant perspective on early Jewish traditions (such as impurity and purity). The following sections will outline the rationale for centering the study of prayer on the body and its physicality.

## Method

During the COVID-19 pandemic, various physical restrictions were imposed on individuals and society to prevent transmission, affecting physical gestures and interactions in interpersonal encounters. Public institutions, organizations, social movements, and various government authorities generated discourse on the role and status of the body in terms of permitted and prohibited activities during this chaotic time.

In response, over the past two years approximately one hundred Jewish prayers have been collected that were created during the coronavirus pandemic. The majority of these prayers were composed by rabbis and religious leaders. Most of these prayers were found on social networks and the websites of major Jewish movements, including the Rabbinical Assembly (Conservative movement) and the Union for Reform Judaism. Prayers written in both Hebrew and English were gathered, and for each prayer, an online link was provided/noted. Furthermore, diversity was ensured by including prayers from various streams within Judaism rather than focusing on just one stream.

This study is based on a textual analysis of five collected prayers, specifically those that were most relevant to the theme—body/embodiment. Textual analysis serves three purposes whereby researchers ascribe meaning to the text, understand the influence of variables outside the text, and critique or evaluate the text (Frey et al., 1992).

By focusing on the structure, verbs, adjectives, metaphors, references, and other liturgical elements within the texts, a range of cultural perspectives are identified concerning the body, self, community, and God. Additionally, the presence of familiar language expressions and traditional prayer patterns is examined, analyzing the interplay between conservatism/preservation and change/renewal. Moreover, the role of divinity within prayer has been delved into. In each category, the prayer itself is provided, accompanied by a comprehensive analysis rooted in sociological and anthropological theories as well as historical writings. The assumption is operated under that these specific texts can shed light on broader social trends and processes.

### Time to be Protected: The Body as a Cooperative Actor in Pandemic Prevention

During the COVID-19 pandemic, frequent washing of the hands became a regular ritual for maintaining hygiene under the assumption that this would prevent infection. This section offers an analysis of two hand-washing prayers: a prayer for cleansing one’s hands and a 20-s prayer during handwashing. Afterward, I discuss another prayer that advances the concept of protecting the body—”A Prayer for Receiving the COVID-19 Vaccine.”

For observant Jews, it is more than just a hygienic practice; it is already a liturgical performance that forms an integral part of their daily routine. In the morning, it is customary to observe the mitzvah of “*netilat yadayim*,” the ritual washing of the hands, immediately after reciting the prayer “*Modeh Ani*” (the morning gratitude prayer)—the prayer recited upon rising. This custom bears resemblance to the practice of priests in the temple, who washed their hands every morning to purify themselves before commencing their work.

According to tradition, there are additional reasons for the obligation of purification through handwashing. First, during the night, it is believed that the hands become impure as they come into contact with impure objects. Second, in the morning, there is a notion that the body carries residual negative energy due to the soul being entrusted to the Creator during sleep. Furthermore, handwashing is also practiced in other situations to maintain purity when not engaged in matters of sanctity, such as after leaving a cemetery (De Sion, 1955, p. 251).^1^

Traditionally, Jews wash their hands and say a blessing before eating meals that include bread or matzah. This ritual, typically done with a two-handled cup, involves pouring water twice on each hand (reversed for left-handed individuals). Despite being unrelated to personal hygiene, it must be performed even if hands are clean. Originating from practices of ritual impurity in the ancient Temple in Jerusalem, where priests had to wash before consuming certain gifts, the rabbis extended this ritual to all Jews to keep the Temple’s practices alive.^2^ Some argue that the practice of washing hands before a meal originates from the dining etiquette of Hellenistic culture (Nadeau, 2010).

In contrast to the preceding examples that highlight the symbolic aspects of purification and impurity in rituals such as handwashing, the COVID-19 prayers introduce a practical dimension to this symbolism by emphasizing the importance of cleansing hands to reduce risk of viral infection. Consequently, the traditional Jewish ritual of handwashing, once imbued with hidden spiritual significance, now assumes a vital role in shaping the emerging behavioral norms, shedding its former esoteric connotations to actively contribute to personal and public health and safety.

Additionally, on a broader conceptual level, these following prayers may challenge the concept of “tum’ah” (impurity)—a state in which a person or object is deemed impure due to specific occurrences, prohibiting that which is impure from approaching anything sacred. While tum’ah traditionally describes a sensory, material, and visual experience, in this case the virus is invisible, yet the writers still invoke rituals of tum’ah, disconnecting from the original idea to convey a new message—personal hygiene. Since these prayers are intended for people who are not Orthodox and may not observe these rituals out of *halachic* knowledge or commitment, conveying the new message can occur more organically and rationally, considering the reality of a pandemic that necessitates adherence to hygiene guidelines.

**Prayer for Cleansing One’s Hands**

Rabbi Lisa Gelber

Holy Eternal One of Life and Love,

Let us balance our well-being between your hands and ours.

May the warm water than runs over my hands, find its way through my fingers and nourish me just as the well of water that followed the people throughout the desert. May the soap that bubbles up from my palms make its way between my fingers, cover the tops of my hands and release the bacteria of fear from my body. May the act of rinsing away soap and water prepare me for the unknown, granting me strength to live with uncertainty. My life is in your hands. Hold me with kindness so I may open my hands to the world even as I keep distance between myself and my neighbor. Bless me with wisdom and good health. May I be safe.

May I be gracious.

May I be grateful.

May I feel whole.

The prayer, written by Rabbi Lisa Gelber, a spiritual leader in Congregation Habonim Community (NYC), affiliated with the Conservative movement, advance the sense of commitment and personal responsibility for cleansing hands. I plea for the water to clean the body is associated with a canonical biblical image – the very well that provided water for the wandering Israelites in the desert.^3^ Thus, the past connects to the present moment of the washing of hands, imbuing it with religious significance and qualifying the ritual.

Continuing with the prayer, there is an emotional confession that arises from the circumstances of fear, uncertainty, and the unknown. These feelings form the core of faith, which is not built solely on clarity and absolute certainty. It is possible that the solution to the situation, which can prevent or mitigate its worsening, lies in practicing careful physical behavior that includes maintaining a safe distance. In this way, medical guidance, given the current circumstances, assumes the significance of a religious mitzvah, serving as a means to establish a dependent relationship between the believer’s health and mental state and the saving power of God.

Thus, the prayer mobilized for the fight to prevent the spread of the virus emphasizes Jewish practices such as handwashing, a tradition rooted in the preservation of bodily health and protection. This ritual, observed after activities like using the bathroom (i.e., the prayer of* Asher Yatzar – “who formed”*) or before meals, takes on heightened significance during the COVID-19 period. As was commonly assumed (especially at the beginning of the epidemic), with the virus transmitted through contact with contaminated surfaces, neglecting hand hygiene can facilitate its spread across different parts of the body.

The second prayer is a 20-Second Prayer During Handwashing, written by Rabbi Joseph Meszler, a spiritual leader of Temple Sinai (Reform congregation) in Sharon (MA).

**A 20-Second Prayer During Handwashing**

Rabbi Joseph Meszler

As I take up my hands

to wash them and

reassure my heart,

I pray for healing and wholeness

for the whole world.

I remember that every life

is unique and of infinite value:

from those living

on the most remote part of the globe

to those in our cities

to our neighbors and family members.

Let me use my hands for good

to help bring love

and compassion to others.

“Let us lift up our hearts and hands

to the Eternal.” (Lam 3:41)

This text also adds that the ritual of handwashing serves as a reminder not only of personal prayer and individual concerns but also of broader human care and responsibility. Indeed, during the COVID-19 pandemic, doctors frequently emphasized the importance of thorough handwashing for at least 20 s with a generous amount of soap. This was because the medical hypothesis suggested that the virus could spread through surfaces.

There is an attempt to recognize all human beings as equals in this global crisis, united in a shared situation, creating a moment to establish empathy and solidarity irrespective of familial or spatial ties. The worshiper seeks empowerment in their hands, desiring the tools to embody the social ideology of love and compassion. They are not passive participants in prayer but active agents of change, possessing the ability to shape reality not only in their minds but also through their own actions.

In Judaism, hands play a central performative role, such as covering one’s eyes during the recitation of the Shema (Deut 6:5), wearing tefillin (phylacteries) or striking the chest during the confessional prayer on Yom Kippur. Meszler chooses to close the prayer with a quote from the Book of Lamentations (3:41), one of the most important documents of ethno-national Jewish trauma and destruction.

In addition to the recent prayers that emphasize the importance of caring for the body and the individual’s responsibility for their health, the following prayer illustrates one of the most significant moments in the interaction between the body and medical discourse: the moment of receiving a vaccination. Amid voices of concern and skepticism regarding the vaccine, the purpose of this prayer, which was composed by Rabbi Naomi Levy, a founder and spiritual leader of Nashuva, a Jewish outreach organization in L.A., is to give thanks for the opportunity to be vaccinated and to bless it.

**A Prayer for Receiving the COVID-19 Vaccine**

By Rabbi Naomi Levy

I have been praying for this day and now it is here!

With great excitement, a touch of trepidation

And with deep gratitude

I give thanks

To all the scientists who toiled day and night

So that I might receive this tiny vaccination

That will protect me and all souls around this world.

With the pandemic still raging

I am blessed to do my part to defeat it.

Let this be the beginning of a new day,

A new time of hope, of joy, of freedom

And most of all, of health.

I thank You, God, for blessing me with life

For sustaining my life

And for enabling me to reach this awe-filled moment.

Amen.

The prayer aims to subvert conspiracy theories and encourage people to embrace vaccination, recognizing the contributions of scientists as agents of healing rather than attributing it solely to a higher power or deity. This approach, characteristic of non-Orthodox denominations, dissolves the tension between faith and science, as the worshiper expresses gratitude toward scientists for their invention of the vaccine through a liturgical tool.

Receiving the vaccine involves dispelling any resistance and unfounded rumors while placing trust in medical expertise to safeguard one’s health. Through prayer, individuals are encouraged to acknowledge their own power and actively participate in preventing the spread of the epidemic by cooperating with their bodies to build immunity. This process not only contributes to scientific knowledge but also fosters social responsibility and self-protection, as well as protecting others. It establishes a sense of responsibility and loyalty toward others by actively monitoring and immunizing the body to maintain good health.

The presence of God is not initially invoked in the creation of the vaccine; instead, God’s role emerges later in the prayer when individuals are granted the discretion to decide whether to be vaccinated. The choice of the noun “awe” may imply a dual meaning: a sense of respect and fear. This alludes to the Days of Awe, which are laden with profound fear, motivating people to confess and seek forgiveness while also showing respect toward others and the forgiving, merciful, and generous nature of God.

### Time to Be Purifying: Reconnecting to a Cleansed Body

In Judaism, water is often associated with the *mikveh* (ritual bath). The water of the mikveh symbolically purifies—it is seen as the water of rebirth. A convert immerses in the mikveh as part of the conversion. Many ultra/modern Orthodox married women go to the mikveh after their menstrual period and before resuming sexual relations. In addition, among some communities, it is common to go to the mikveh before marriage, as do many men before Yom Kippur, with some going every Friday before Shabbat. The mikveh can be any source of water that is considered “living water”—water obtained naturally—through rain, rivers, springs, lakes and oceans; that is, water that can support life. Although the use of the mikveh has changed over time, its purpose has not changed. It always prepared Jews to enter moments of holiness, preceding the holy entry into marriage, conception, Judaism, and holy days.

Today, the mikveh is also used to mark the differences between strict Halakhic observances, mere customs and modern and liberal rituals. For instance, according to Crasnow (2017), for some trans Jews, the moment of liminality encountered at the mikvah may couple in meaningful ways with liminality in their own lives, mirroring the experience of living in the unstructured or in-between spaces of non-normative gender and sexuality. This may be experienced as permanent marginality for those whose genders are not binary or temporary liminality for those who are transitioning across binary gender (2017: 407).

This rich history of Jewish immersion inspired Rabbi Hannah Estrin, from the Congregation of Moses in Kalamazoo (Michigan), to compose a post-COVID-19 Mikveh Ceremony. In the introduction to the prayer, she explains:

“Historically, we have seen water as a creator of significant changes in the world and life. Creator of a new normal. From the story of creation to the birth of a child and eventually the washing of a body following death, water surrounds our entire lives. It is the powerful flash flood, the gentle lapping wave, the all-encompassing embrace of submersion. Water is in our life force and is a symbol of renewal for many people around the world. We can harness some of the power and beauty of water to intentionally create our path forward. I offer this mikveh ceremony for those who are ready to mark their transition toward a post-COVID-19 new normal. We are entering a new phase that will hold both joy and hope as well as sadness. We have been forever changed, individually and communally. However, we are strong and resilient. A visit to the mikveh or local “living source” of water can be a powerful step in that process”.

This particular prayer, including the instructions, provides an opportunity for introspection and sincere self-reflection, even prior to reaching the mikveh. It does not depict a utopian past or dystopian reality. Rather, it describes the present reality in its most current form, acknowledging the challenges and hardships faced by the individual, including the experience of physical and bodily isolation. Simultaneously, prayer fosters an appreciation for oneself and the supportive environment that surrounds the believer.

The act of ritual immersion not only serves to purify the individual from a challenging period but also serves as the gateway to community reintegration and full participation. It represents a meditative moment, a practice of mindfulness that centers on the present, the here and now, while simultaneously connecting with the divine through the transformative power of the mediating water. There is significant emphasis placed on the experiences of distant bodies, those that remain separate and untouched, which can generate feelings of loneliness and alienation within the believer.

In addition, its traditional text, Rabbi Estrin’s prayer, also incorporates the traditional prayer of immersion. Furthermore, there is a suggestion for a spontaneous and personal prayer that may not adhere strictly to the prescribed or traditional text but rather reflects the creativity and authentic thoughts that arise within the individual in that performance. The concluding text of the prayer carries an optimistic tone, reinforcing the individual’s empowerment and shared responsibility with God in shaping a transformed social reality.

**Post COVID-19 Mikveh Ceremony**

by Rabbi Hannah Estrin

לַכֹּ֖ל זְמָ֑ן וְעֵ֥ת לְכָל־חֵ֖פֶץ תַּ֥חַת הַשָּׁמָֽיִם׃

“A season is set for everything, a time for every experience under heaven” —Kohelet

Everything has a time

A time for staying home and a time for physical distancing.

A time for masks and gloves and protections.

A time for buildings, parks and beaches to be closed.

There is a time for life to happen online. And a time to remain apart.

This does not mean that we have returned to life as it was. We cannot. The world has changed and we have changed with it. Immersing in the waters of life is a way to mark the sacredness of time. A way to move forward in creating a new normal. A step toward rejoining the physical community.

I want to name and honor my reactions during this difficult time.

To know that my reactions, though different from those around me, were OK.

To recognize that my trauma and grief are real and honest. That each of us is different.

To honor the challenges, I have experienced and those I may continue to have.

By honoring all that has been, I choose to let go and move forward. To acknowledge anew my trust in God, and God’s control. With that, I pray to be open to my return to life as it will be and all the opportunities it will present.

**If there are others who are joining you for your immersion:**

Each of you has been a blessing to me during this time of difficulty. For your place in my life, for your lovingkindness and support, I thank you and offer you my love.

**Entering the mikveh/beach**

Recite at the mikveh steps:

As I step toward these waters of healing and preparation, I recognize the period where my life was turned upside down by outside forces. Where some days were easier than others but through it all I underwent a transition from who I was to who I will become. As I prepare to step into the healing waters of the mikveh I choose also to let go. I allow it to envelope me in a way that only the waters of God can. To cradle and hold me as I prepare to step back into life. I remind myself that though people have been physically distant, I am always loved, held, and protected. That I am strong and I will be OK.

בָּרוּךְ אַתָּה אֲדֹנָי אֱלֹהֵינוּ מֶלֶךְ הָעוֹלָם אֲשֶׁר קִדְּשָׁנוּ בְּמִצְוֹתָיו וְצִוָּנוּ עַל הַטְבִלָה

*Barukh atah Adonay Eloheynu melekh ha-olam, asher kidshanu b’mitzvotav v’tzivanu al ha-t’vilah.* Blessed are you, Eternal God, ruler of the universe, who sanctifies us through *mitzvot*

בָּרוּךְ אַתָּה אֲדֹנָי אֱלֹהֵינוּ מֶלֶךְ הָעוֹלָם, שֶׁהֶחֱיָנוּ וְקִיְּמָנוּ וְהִגִּיעָנוּ לַזְּמָן הַזֶּה

*Barukh atah Adonay, Eloheynu melekh ha-olam, shehekheyanu, v’kiy’manu, v’higianu, la-z’man ha-zeh.*

Blessed is the Eternal, the God of all creation, who has blessed me with life, sustained me, and enabled me to reach this moment.

**As you exit the mikveh:**

Adonai, my God who heals the brokenhearted and restores the body, I stand here before you. My heart has been broken and my pain is profound. I look to you for healing and strength as I return to the world we build together. I know not what tomorrow will bring, but place my trust and my life in your hands. Together. You and I together with community and the world. May my days be filled with preserving the past and creating the future filled with your guidance, love, tolerance, peace and humility. Amen.

### Time to be Pregnant? Praying for a Fertile Body

Observing fertility goes beyond confirming a biological matter; it provides an opportunity for a socio-cultural examination of the individual within society, especially among minorities such as the Jews (Irshai, 2012; Okun, 2017; Raucher, 2021). Drawing on an ethnographic study of reproduction in Israel, Taragin-Zeller (2019) demonstrates how Orthodox Jews delineate borders between the godly and the human in their daily reproductive practices. Berkovich (1997: 605) argued that the construction of a distinct category of women that emphasizes women’s difference takes place within an ideological context of the self-conscious myth of gender equality. Motherhood is defined as a public role that carries national significance.

With the outbreak of the coronavirus in its initial stages in Israel, fertility treatments were halted due to concerns about the medical teams contracting the virus. However, on May 11, 2020, the Ministry of Health of Israel approved the resumption of these treatments. Before the decision was made, Ziva Monsongo, an Orthodox Israeli woman, composed a Prayer for Halting Fertility Treatments. This prayer gained widespread distribution on the Internet (see Table 1).

**Table 1:** Prayer for halting fertility treatments

Open a gate for us When the gate is locked In the shadow of the plague Fertility treatment gates were closed The heart of many couples is broken! The anticipation and hope in the moment of waiting From the darkness To our father in heaven, we offer a prayer: Waiting will bring a gift Please, God, salvation please Open a woman’s womb longing, expecting A small and sweet fetus will be received and will fill a deep space The day will turn The sun will rise and set We will enter your gates To offer the new mother’s sacrifice	פְּתַח לָנוּ שַׁעַר בְּעֵת נְעִילַת שַׁעַר בְּצֵל הַמַּגֵּפָה נִנְעֲלוּ שַׁעֲרֵי טִפּוּלֵי הַפּוֹרִיּוּת !לִבָּם שֶׁל זוּגוֹת רַבִּים נִשְׁבָּר הַצִּפִּיָּה וְהַתִּקְוָה בְּרֶגַע שֶׁל הַמְתָּנָה מִתּוֹךְ הָאֲפֵלָה :לְאָבִינוּ שֶׁבַּשָּׁמַיִם נִשָּׂא תְּפִלָּה הַהַמְתָּנָה תָּבִיא מַתָּנָה אָנָּא ה’ הוֹשִׁיעָה נָּא פְּתַח רֶחֶם לְאִשָּׁה כְּמֵהָה, מְצַפָּה יִקָּלֵט עֻבָּר זָעִיר וּמָתוֹק יְמַלֵּא חָלָל עָמֹק הַיּוֹם יִפְנֶה הַשֶּׁמֶשׁ יָבֹא וְיִפְנֶה נָבוֹאָה שְׁעָרֶיךָ לְהַקְרִיב קָרְבַּן יוֹלֶדֶת

The choice to begin the prayer with “Open a gate for us,” a familiar verse from the closing service of Yom Kippur (and based on Psalm 118), brings the worshiper back to one of the most significant moments in the Jewish annual prayer cycle (Hoffman, 2018). It represents the final and desperate plea of the worshiper just before the closing of the gates of Heaven. However, in this context, it is not about the woman’s personal sins. She has not committed any wrongdoing, nor is she responsible for the decision of the country’s medical services to close fertility centers. Nevertheless, the prayer carries a tone of guilt, emphasizing the notion that any attempt to hinder the advancement of the Jewish population in Israel is indeed a sin.

Therefore, there is a direct plea to God to change the perilous reality, not by intervening in the decision-making process of elected officials and policymakers but by intervening in the woman’s body itself, as she waits and yearns for the moment of conceiving. The purpose of the fetus is to fill a deep void within the woman’s body, so as long as she is not pregnant, there remains an empty cavity, an abnormal state that reflects the spirit of the times. This statement reflects a prevailing public ideology in Israel of natalism (Donath, 2011).

This prayer demonstrates the importance of fertility matters in Israel, but not only that, it also reflects and at the same time—by virtue of the fact that it was created—also establishes the perception of the Israeli Jewish woman first of all as mother and woman and not as individual or citizen. This may explain the author’s poetic choice to end the prayer with a parallel between the work of temple worship (offering the sacrifices) and the commandment of procreation. Furthermore, following Avishai, Jafar and Rinaldo (2015:5) who suggest that gender and religion as mutually constitutive social categories”, this particular text can thus reinforce the importance of female liturgy (Falk, 1987; Marx, 2009), grounded in women’s physical, family, and social life experiences, as well as the ritual efficacy of Jewish women (Brown, 2023a).

Furthermore, as a collective prayer, the community functions as a supportive and encouraging framework in the situation at hand, aiming to provide optimism and acknowledge the patience required by society for the eradication of the pandemic. This is reflected, for example, in the choice to phrase the prayer in the plural language rather than the singular. Thus, it is not a personal plea from the woman to God, but a collective supplication from the community on behalf of the woman and the couples yearning to conceive.

### Time to Die: Mourning the Dead Body

In the Jewish world, the attitude toward the sick body is accompanied by ambivalence. On the one hand, the value of the sanctity of life (*Kedushat HaChaim*) requires avoiding contact with the patient to prevent the spread of disease. On the other hand, visiting the sick and caring for an individual’s health is considered a *mitzvah* that fosters interpersonal and community interaction. The moment when physical distance from the ‘other’ becomes permanent is death. This moment also serves as a reflection for the living, highlighting the fragility of the body and the transient nature of life. Sometimes, we take life for granted and suppress these thoughts daily. Jewish liturgy is employed to address this anomaly, with rituals and orderly rules guiding the management of burial and mourning periods (such as sitting shiva^4^) (Davis, 2008; Ochs, 2017).

Hazan and Gamliel (2003) demonstrate the enduring significance of burial and funeral rituals, steeped in religious and ecstatic symbols. These rituals emphasize a dichotomy between body and soul, challenging the simplistic view of the body as mere flesh and blood. Instead, they focus on the departed spirit’s transition to the world of the afterlife, reinforcing the importance of ‘respect for the dead.’ This concept arises from the belief in the complete separation of body and soul at death, as well as societal perceptions of the human body—an argument that extends back to the days of the Pharisees and the Saducees two thousand years ago.

“Dignity in Death” is a concept embodied in funeral and burial ceremonies, representing rites of passage and crisis. These rituals navigate the transition between life and death, creating a bridge during the liminal phase when the deceased is neither considered alive nor fully assimilated into the world of the deceased. Underlying this notion is the understanding that society is a network of relationships among individuals. When a person dies, society does not merely lose a single member; it loses part of its collective identity, shaking its self-belief. In this context, these rituals serve to preserve the community by reviving its values and reconnecting severed relationship threads. The public presence of the deceased challenges society’s resilience and the determination of the deceased’s position, with their dishonor reflecting upon the community’s dignity.

During the COVID-19 period, due to restrictions on gatherings, funerals were held in very limited frameworks and sometimes moved to Zoom, similar to other life-cycle rituals. The restrictions posed a new challenge to family members and relatives who consider the funeral ceremony’s conduct very important (Khosa-Nkatini & White, 2021). This meant that the body of the deceased was distanced from the participants who would normally have come to pay their last respects on such occasions.

For example, Bitusikova (2020) contends that the pandemic has prompted a reevaluation of our relationship with death, encouraging people to explore creative approaches to end-of-life practices. In her research, she participated in a Jewish funeral conducted via Zoom and found that this virtual format provided a unique opportunity to visually showcase the deceased’s life, including their significant achievements and milestones, to distant relatives and friends who may not have known them well. This online ceremony became celebratory and widely shared tribute to the person’s life. Bitusikova suggests that the virtual experience was remarkably personal, emotional, and intimate, possibly due to the close-up view of participants’ faces and their homes (54).

The following prayer, composed by Rabbi Naomi Levy is intended for funerals. It symbolizes, in the most ritualistic manner, the moment of disconnection from any physical contact or sight with the body of a deceased person during the COVID-19 period.

**A Prayer for a Funeral from Afar**

by Rabbi Naomi Levy

It breaks my heart

To lay you to rest this way

From a distance

Without the sendoff you deserve.

No touch, no kiss, no loving crowd in attendance.

However, I know your soul has already moved on

From the body we bury today

To the place of eternal knowing and peace

In God’s shelter.

I can’t bear your casket

But I can bear witness

To your undying legacy:

I miss you.

And nothing –

No person, no joy, no accomplishment, no distraction,

Not even God

Can fill the gaping hole your absence has left in my life.

But mixed together with all my sadness,

There is a great joy for having known you.

I want to thank you for the time we shared,

For the love you gave,

For the wisdom you spread.

Thank you for the magnificent moments

And for the ordinary ones too.

There was beauty in our simplicity

Holiness in our unspectacular days

And I will carry the lessons you taught me always.

Your life has ended

But your light can never be extinguished.

It continues to shine upon me

Even on the darkest nights

And illuminates my way.

May God watch over you and bless you

As you have blessed me

With love, with grace

And with peace,

Amen.

In contrast to previous prayers centered around the body, which highlighted the functions and essentiality of its organs, this particular prayer places the body in a state of present-absence, aligning with the context for which it was crafted. The prayer does not explicitly reference the body but instead focuses on the individual who was once present and is now absent. This nuanced approach is noteworthy as it unveils the human perception of the deceased, portraying them as a formless entity acknowledged only at the conclusion, emphasizing their absence.

The prayer begins with a poignant emotional confession, reflecting the difficulty of holding a funeral without the physical presence of participants. This confession and sense of longing continue into the second stanza of the prayer. The speaker in the prayer acknowledges the binary concept of body and soul (Novak, 2002), understanding that the soul has already transitioned to another place. This concept helps to recognize a form of continuity and eases the cognitive challenge of grasping the departure of the departed. In this anguished prayer, there is a radical statement that suggests that God’s intervention is not required to rectify the situation or to alleviate the difficulty of comprehending the anomalous moment: *“Not even God can fill the gaping hole your absence has left in my life”.*

Indeed, this prayer encompasses not only sadness but also a profound acknowledgment and gratitude for the meaningful relationship shared with the departed. The memories continue to reverberate, and the belief that they will forever endure serves as a source of solace in coping with the loss. Furthermore, there is a specific request addressed to God to bless the deceased, as if they were still alive, without any explicit mention of the afterlife or heaven. This, similar to the pessimistic statement mentioned earlier that suggests that not even God can succeed in restoring a broken soul, contrasts with other funeral prayers in Judaism, which often refer to the soul’s journey to the next world. This unique aspect of the prayer highlights the focus on the enduring impact of the deceased’s life on those left behind, emphasizing the preservation of their memory in this world.

Thus, this prayer engages with and reflects non-Orthodox Jewish theological perspectives on commemoration and memory,^5^ focusing on death, the individual’s personality, and life story, while acknowledging those left behind who seek to continue their lives and remember the deceased.

## Conclusion

*“Everything has an appointed season, and there is a time for every matter under heaven”.* In these words, Ecclesiastes (Kohelet, Chapter 3) expresses the notion that every event in a person’s life has its own appointed time. Even so, prayer serves as a strategic response to adapt and adjust to new realities. COVID-19 prayers are thus spiritual, social and cultural tools to face illness, sorrow, and uncertainty—as well as a channel to convey medical behavioristic messages.

Through these prayers, we gain insights into how various religious communities cast a unique spotlight on the individual and social perception of the human body: such as the call for cleanliness, vaccination, spiritual connection, purification, or bodily imperfections. During the COVID-19 period, the body was often viewed as a source of contagion due to non-compliance with preventive measures. However, in these texts, the body is portrayed differently—it emerges as a compliant and disciplined agent.

Thus, the prayers aim to restore harmony in the individual’s relationship with their body and with others. Through prayer, the body is regarded as requiring cleansing, vaccination, and purification, resulting in the continuation of its functions. The concern for a healthy body thus translates into concern for society. Indeed, these values can range from universal social ideals such as the emphasis on science over faith to specific missions such as the promotion of mitzvot (for example, fertility) or the enduring commitment to faith (manifested in prayers that recognize and extol the power of the saving God).

As instrument and symbolic social agent, a theological view of the body is validated in which liturgical texts become mediators not only for the worship of God, but also for recognition of the individual and affirmation of the body. This tendency is well reflected in prayers that place the individual at the center of responsibility for eradicating the pandemic by adopting protective behavioral codes (such as handwashing or vaccination), changing traditional rituals (such as funerals), and adopting new rituals to connect with the bodies of the sick (for example, immersion in the mikveh). The body is under the individual’s authority, and their duty is to affirm their own body’s existence and that of others bodies.

In particular, non-halakhic Jewish liturgies, particularly the Reform Judaism, respect the sovereignty of the worshiper over their body, in contrast to halachic Judaism. In these texts, we find the individual as a central agent in prayer, bearing the responsibility of eradicating the pandemic and caring for others. Additionally, talented individuals, namely, scientists, will provide medical solutions rather than relying on a miracle from heaven. Furthermore, there were also expressions of discontent directed toward God, attempting to humanize Him, and criticism regarding the overwhelming grief caused by the deaths of the pandemic’s victims, suggesting that even God Himself cannot comprehend the magnitude of the suffering.

The content of the prayers not only places God at the center, expressing appreciation and gratitude toward Him (as noted by Heschel, 1954), but also centers the individual and their body, praising not only God but also scientists and doctors. Additionally, contrary to Derrida’s argument that prayer involves a willingness to relinquish the expectation of a response (2013), these prayers do indeed reflect an expectation that the request will be fulfilled; both the individual’s request to adhere to the behavioral guidelines for eradicating the pandemic and the request to God to eliminate the virus.

Therefore, I propose viewing this as a “theology of humanistic responsibility,” one that places the individual’s life at the center—not only within the social order but also within the prayer itself—while enlisting God, religious practices, and faith to sanctify this goal. This approach does not dismiss God in response to the difficult emotions that may arise in individuals who feel like helpless victims in a chaotic reality. Rather, it turns to Him, recognizing His power and responsibility to change the situation, while simultaneously acknowledging the roles of the patient and the doctor in combating the pandemic.

This may provide insight into the perspectives of the prayer authors and illuminate their dynamic relationship with Jewish tradition, including the ideas it embodies and the various texts and practices it offers. It could reveal a bidirectional movement toward and away from tradition; on one hand, using familiar traditional rituals (such as handwashing or immersion in a mikveh, for example) but with innovative conceptual changes adapted to the current context, thereby validating the act.

Following the historical discussion on Jewish responses to epidemic outbreaks and the general population’s biased attitudes toward Jews often blamed for spreading diseases (Brown, 2023b), these prayers can be viewed as endorsing, on one hand, the Talmudic reading that epidemics are creations of the Creator, hence the appeal to Him to stem them. Additionally, they track stereotypical reactions toward Jews, such as accusations directed at the ultra-Orthodox community for spreading the Corona virus (Gilman, 2021), as these prayers demonstrate efforts to combat the epidemic and promote adherence to guidelines to prevent transmission.

Furthermore, the prayers also reflect medical assumptions and provisional diagnostics obtained during the epidemiological investigation of the virus’s spread. For instance, at one juncture, research indicated that there surface transmission played no or negligible role in spreading SARS-CoV-2 (Pilipenco et al., 2023). Consequently, it can be inferred that these prayers do not solely rely on empirical scientific sources but rather respond to temporary diagnoses. Therefore, it is plausible to suggest that once these first diagnoses are found incorrect, the relevance of the prayers may diminish, much like the diagnoses themselves.

In sum, the very creation of these prayers demonstrates the central place of Jewish tradition in providing a response during times of crisis and into the post-coronavirus era. Follow-up studies, both qualitative (Chukwuma, 2021) and quantitative (Sánchez-Camacho & Martínez, 2021), should explore how COVID-19 prayers were experienced by the worshipers themselves. Indeed, prayer is not solely a written text; it is also a personal and phenomenological experience deeply rooted in the individual’s world. This article aimed to focus solely on the written aspect and did not delve into the performative dimension of the performance. Therefore, interviews with the prayer creators and worshipers themselves will help establish a connection between liturgy/theology and sociology.

## Footnotes

1 For an historical survey of this custom, see Meir Benayahu, “The Evolution of Customs Rooted in the Concept of Ruah HaTumʾah,” in M. Benayahu, Studies, 305-12, esp. 305-7.

2 See: Talmud Bavli, Shabbat 14a.

3 The Holy One, blessed be He, said: If I lead them into the land by the most direct route, they will each take possession of a field and a vineyard and neglect the law. Therefore, I will lead them through the wilderness for forty years and cause them to eat the manna and drink the water of the well so that the law may penetrate into their very beings (Midrash Tanchuma, Beshalach 1).

4 According to tradition, following the burial of a deceased individual, their relatives are obligated to observe mourning customs for a duration of seven days, starting from the day of the burial.

5 For example, within Reform Judaism, there is a spectrum of views on assisted dying, yet there is consensus on the importance of dignity in death for all individuals, even amid varying interpretations shaped by personal experiences, theological beliefs, and research. Unlike certain sectors of the Jewish community where ritual sometimes supersedes the needs of mourners, Reform communities strive to support mourners in ways that feel most meaningful to them, striking a balance between traditional Jewish values of Kavod HaMet (respect for the deceased) and Kavod HaChayim (respect for the living) (Romain, 2014).
