# Correction to: “With the Pandemic Still Raging, I am Blessed to Do My Part to Defeat it”: Exploring COVID‑19 Jewish Liturgy and Prayers in Israel and the United States

**DOI:** 10.1007/s10943-024-02241-y

**Published:** 2025-02-07

**Authors:** Elazar Ben‑Lulu

**Affiliations:** Ariel University, Ariel, Israel

**Correction to: Journal of Religion and Health** **https://doi.org/10.1007/s10943-024-02190-6**

In this article, the Hebrew prayers written as left to right incorrectly instead of right to left. The original article has been corrected.

The prayer under the heading “**Post COVID-19 Mikveh Ceremony**” was incorrect. It should have been appeared as given below.

**Incorrect:**



**Correct:**



The prayer under the heading, “**Entering the mikveh/beach**” was incorrect. It should have been appeared as given below.

**Incorrect:**



**Correct:**



**Incorrect:**



**Correct:**



In Table 1, the prayer should be written in right to left . The correct version of Table 1 is given below.

**Incorrect version of Table 1:**

**Table 1:**
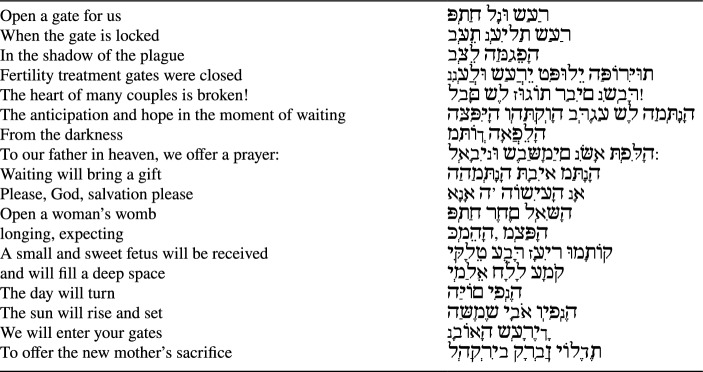
Prayer for halting fertility treatments

**Correct version of Table 1:**

**Table 1:**
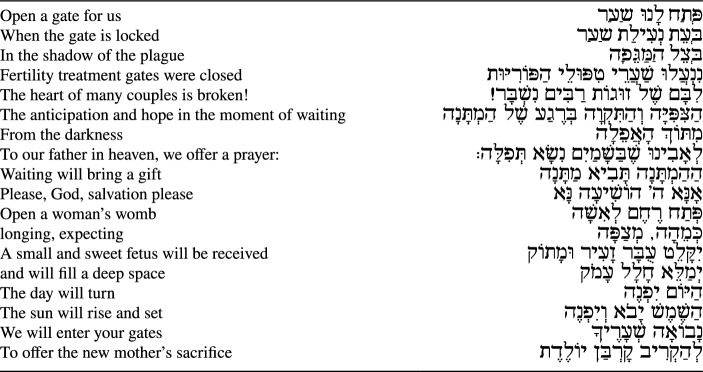
Prayer for halting fertility treatments

